# A Novel System for Precise Grading of Glioma

**DOI:** 10.3390/bioengineering9100532

**Published:** 2022-10-07

**Authors:** Ahmed Alksas, Mohamed Shehata, Hala Atef, Fatma Sherif, Norah Saleh Alghamdi, Mohammed Ghazal, Sherif Abdel Fattah, Lamiaa Galal El-Serougy, Ayman El-Baz

**Affiliations:** 1 Bioengineering Department, University of Louisville, Louisville, KY 40292, USA; 2 Department of Radiology, Faculty of Medicine, Mansoura University, Mansoura 35516, Egypt; 3 Department of Computer Sciences, College of Computer and Information Sciences, Princess Nourah Bint Abdulrahman University, P.O. Box 84428, Riyadh 11671, Saudi Arabia; 4 Electrical, Computer, and Biomedical Engineering Department, Abu Dhabi University, Abu Dhabi 59911, United Arab Emirates

**Keywords:** GG-CAD, MRIs, HOG, GLCM, GLRLM, ADC

## Abstract

Gliomas are the most common type of primary brain tumors and one of the highest causes of mortality worldwide. Accurate grading of gliomas is of immense importance to administer proper treatment plans. In this paper, we develop a comprehensive non-invasive multimodal magnetic resonance (MR)-based computer-aided diagnostic (CAD) system to precisely differentiate between different grades of gliomas (Grades: I, II, III, and IV). A total of 99 patients with gliomas (M = 49, F = 50, age range = 1–79 years) were included after providing their informed consent to participate in this study. The proposed imaging-based glioma grading (GG-CAD) system utilizes three different MR imaging modalities, namely; contrast-enhanced T1-MR, T2-MR known as fluid-attenuated inversion-recovery (FLAIR), and diffusion-weighted (DW-MR) to extract the following imaging features: (i) morphological features based on constructing the histogram of oriented gradients (HOG) and estimating the glioma volume, (ii) first and second orders textural features by constructing histogram, gray-level run length matrix (GLRLM), and gray-level co-occurrence matrix (GLCM), (iii) functional features by estimating voxel-wise apparent diffusion coefficients (ADC) and contrast-enhancement slope. These features are then integrated together and processed using a Gini impurity-based selection approach to find the optimal set of significant features. The reduced significant features are then fed to a multi-layer perceptron artificial neural networks (MLP-ANN) classification model to obtain the final diagnosis of a glioma tumor as Grade I, II, III, or IV. The GG-CAD system was evaluated on the enrolled 99 gliomas (Grade I = 13, Grade II = 22, Grade III = 22, and Grade IV = 42) using a leave-one-subject-out (LOSO) and *k*-fold stratified (with *k* = 5 and 10) cross-validation approach. The GG-CAD achieved 0.96 ± 0.02 quadratic-weighted Cohen’s kappa and 95.8% ± 1.9% overall diagnostic accuracy at LOSO and an outstanding diagnostic performance at *k* = 10 and 5. Alternative classifiers, including RFs and SVM${}_{lin}$ produced inferior results compared to the proposed MLP-ANN GG-CAD system. These findings demonstrate the feasibility of the proposed CAD system as a novel tool to objectively characterize gliomas using the comprehensive extracted and selected imaging features. The developed GG-CAD system holds promise to be used as a non-invasive diagnostic tool for Precise Grading of Glioma.

## 1. Introduction

In 2022, brain and spinal cord primary cancerous tumors will be diagnosed in an estimated 25,050 adults in the United States (14,170 male and 10,880 female). Despite the odds of developing these tumors in one’s lifetime being less than one percent, they represent 85% to 90% of all primary central nervous system (CNS) tumors. With a high mortality rate among adults in the USA (18,280 deaths estimated in 2022), cancer of the CNS is the 10th leading cause of death [1]. Glioma is the most common primary malignant tumor of the CNS in adults, demonstrating distinct characteristics. It represents the highest prevalent cerebral tumor, which takes place in the cerebral glial tissues comprising nearly 30% of brain tumors. Its incidence is about 5–10 per 100,000 people every year with serious morbidity and high mortality rates [2]. Despite improvements in medical treatment targeting glioma-specific molecular pathways, the prognosis remains poor. The median survival is less than 15 months following primary diagnosis, and the five-year survival rate is less than 10% [3,4,5]. The tendency of malignant gliomas to infiltrate the brain parenchyma in a diffuse manner and the particular tumor microenvironment, that may promote glioma development, contribute to their resistance to conventional surgical therapy and other treatment strategies.

According to the classification of the World Health Organization (WHO) [6,7], gliomas are categorized into four grades based on cellular morphology and malignant tumoral behavior. Grade I gliomas are biologically benign with low risk, while WHO Grade II gliomas are considered low-grade gliomas, with benign tendency, however, they have a considerable recurrence rate. WHO Grade III glioma (anaplastic glioma) and Grade IV glioma (glioblastoma) are considered high grade undifferentiated malignant gliomas, with poor prognosis [2,8]. The most aggressive, glioblastoma, has a survival rate of only 6.8% [9]. Gliomas are graded primarily on the histopathology of tissue obtained through surgical biopsy or resection, which shows the malignancy scale of the tumor. Revised criteria for glioma grading were published with the 2016 revision of the WHO classification, taking into account molecular and genetic information along with the histological features of the tumors, aiming at better prediction of tumor behavior, treatment response, and prognosis [10,11,12]. Precise tumor grading is of immense importance for guiding neuro-oncologists to the proper decisions for tumor treatment planning and consequently better patient prognosis [13,14]. Despite the fact that the biopsy is the reference standard for identifying the grade of gliomas, it is not favorable because of high invasiveness, expense, and its adverse effects such as bleeding and infection. Therefore, many researchers were motivated to investigate imaging techniques for a non-invasive, early, and precise grading of gliomas for a timely management plan [15,16,17,18,19,20,21,22,23,24]. In particular, magnetic resonance imaging (MRI) is the most common imaging modality for the diagnosis and assessment of cerebral neoplasms, including gliomas. Conventional MRI can evaluate the anatomy of the tumor regarding location, morphology, multiplicity, and mass-related effects. Advanced MRI submodalities such as diffusion- and perfusion-weighted imaging, as well as magnetic resonance spectroscopy (MRS), have the ability to provide physiological information of brain tumors. They can provide quantitative metrics of tumor cellularity, vascularization, and metabolism before and after treatment [12,25,26,27]. Particularly, diffusion-weighted MR (DW-MR) has proven its ability to differentiate gliomas by grade, and in determining the prognosis. In general, higher grades are related to reduced apparent diffusion coefficient (ADC), as water diffusion is substantially decreased in the setting of increased tumor cellularity [14]. Perfusion MR imaging techniques including arterial spin labeling (ASL) assess tumor vascularity associated with neoangiogenesis [27]. MRS gives information about the tumor internal biochemical milieu, by assessment of the concentration of different tumoral cell metabolites. In glial brain tumor grading; N-Acetyl Aspartate (NAA) is a neuronal integrity marker, choline (Cho) a cell membrane breakdown and turnover marker while creatinine (Cr) is a metabolism marker. Increased glioma grade is related to increased aggression of tumor cells that is associated with rising in Cho and reduction in both NAA and Cr levels as well as increased Cho/NAA and Cho/Cr ratios [28].

With the recent advances in artificial intelligence, specifically, machine learning algorithms, and the important role they are playing in the early diagnosis, detection, and prediction of different medical conditions [29,30], they have been incorporated in different studies to help with the clinical diagnosis of gliomas [31]. For example, Zhang et al. [16] explored the abilities of texture analysis along with machine learning on multimodal MR images to differentiate between glioma grades. Their study included a total of 120 subjects with gliomas, 28 low-grade gliomas (LGGs) and 92 high-grade gliomas (HGGs) (Grade I = 3, Grade II = 25, Grade III = 29, and Grade IV = 63). They extracted texture features from the aforementioned data and then performed an oversampling technique to balance their data. In their study, a total of 25 machine learning classifiers were incorporated for classification purposes. The optimal classification model achieved the highest accuracy of 96.1% in grading gliomas (Grades: II, III, and IV) using oversampling, while achieving 78.6% accuracy on the original data. Despite their promising results, their model did not investigate any functional or appearance features which could have led to higher performance especially with the unbalanced data. Another recent study by Cho and Park [17] assessed the accuracy for classification of gliomas by characterization of multimodal MRI data from the MICCAI Brain Tumor Segmentation Challenge (BRATs 2015) [32]. They utilized the logistic regression using shape and texture features to classify gliomas (LGG = 54 and HGG = 54). They reported an overall accuracy of 89.8%. Suarez-Garcia et al. [18] investigated the role of multimodal-MRI along with texture features to identify HGG from LGG. A total of 285 subjects were obtained (HGG = 210 and LGG = 75) from the BRATs 2018 challenge. They extracted texture features from the gray level size zone matrix. These features are then fed to multiple linear regression models toward finding the best model to classify gliomas. Their best model was reported to have an accuracy of 91.8% distinguishing HGG from LGG. Banerjee et al. [19] evaluated the abilities of CNN along with multi-sequence MR images to differentiate HGG from LGG. The authors included 746 patients (HGG = 472 and LGG = 274) from The Cancer Imaging Archive (TCIA) [33] and BRATs. The study proposed two fine-tuned ConvNets and evaluated these models using leave-one-subject-out. They reported their highest accuracy of 95% for classification of gliomas. In another recent study, Alis et al. [20] utilized the artificial neural networks (ANN) to differentiate HGG from LGG. The authors assessed the diagnostic accuracy of conventional MR images texture analysis. They extracted first and second order texture features from manually-placed ROIs of 181 subjects (HGG = 97 and LGG = 84). Their proposed pre-trained ANN achieved an accuracy of 88.3% discriminating HGG from LGG. Hsieh et al. [21] evaluated intensity-invariant MR imaging obtained from 107 patients diagnosed with glioma (HGG = 34 and LGG = 73). They extracted texture features and histogram moments from local binary patterns of manually-delineated tumors. These features were then combined in a binary logistic regression classifier to distinguish HGG from LGG. They reported an accuracy of 93%. Six CNN models were developed by Kalaiselvi et al. [22] for the classification of glioma lesions from volumetric MR scans. Their study included 4500 images extracted randomly from BRATs 2013 (30 volumes) and Whole Brain Atlas (WBA) (8 volumes). Their best model successfully discriminated HGG from LGG with an accuracy of 88.91%. Zhuge et al. [23] gathered conventional MR images of 315 patients (HGG = 210 and LGG = 105) from TCIA and BRATs 2018 to develop two deep learning models for distinguishing HGG from LGG. With an accuracy of 97.1%, their proposed 3D ConvNet model achieved the best diagnostic performance. Using multimodal MR images of 285 cases (HGG = 210 and LGG = 75) from the BRATs 2017, Cho et al. [24] assessed the diagnostic performance of radiomic features for discriminating between LGG and HGG. The authors extracted 468 radiomic features and then they selected their optimal features to build three different machine learning classification models. They reported an average accuracy of 88.54% using three different classifiers: logistic regression, SVM, and random forest classifiers.

Despite the fact that these papers represent the state-of-the-art research studies for grading gliomas using MRIs along with computer-aided diagnostic (CAD) systems, they had some limitations that need to be addressed. Most of these studies [17,18,19,20,21,22,23,24] only focused on differentiating between LGG and HGG instead of grading these tumors into Grades I, II, III, or IV for a proper management plan. In addition, none of the aforementioned studies incorporated any functional features for a better diagnostic performance. Some of the authors [16,18,20,21] incorporated texture features in their work, but they did not investigate any shape or appearance features which could have led to higher performance as well.

**Figure 1:**
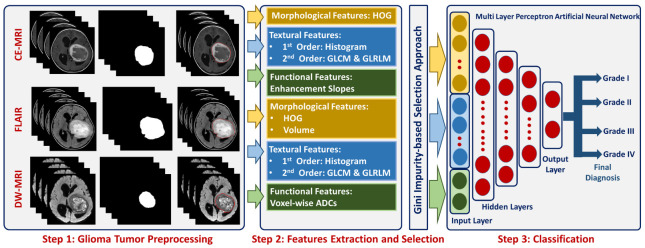
The developed GG-CAD system for accurate grading of glioma tumors.

To circumvent for these drawbacks, we introduce a novel comprehensive glioma grading CAD (GG-CAD) system, shown in Figure 1. To the best of our knowledge, the GG-CAD system is the first of its kind to integrate novel 3D appearance features, volumetric features, 3D first and second order textural features, with functional features extracted from multimodal MR images to precisely identify the glioma grade as Grade I, II, III, or IV for a proper medical management plan.

## 2. Materials

Input Data Description: A total of 99 biopsy confirmed glioma tumors (M = 49 and F = 50) with age range between 1 and 79 years and average of 40.15 ± 19.94 years were included (Grade I = 13, Grade II = 22, Grade III = 22, and Grade IV = 42). All patients, themselves or their parents/legal guardians (for minors with age < 18 years), provided their informed consent to participate in this study. Multimodal MR images, namely; T1-MR with pre- and post-contrast phases, T2-MR (FLAIR), and DW-MR, were acquired at Mansoura University Hospital, Egypt.

Imaging Protocols: The MRI examinations were performed using a 1.5 Tesla scanner (Ingena, Philips medical system, Best, The Netherlands). Examination was done with the participants in the supine position, using a standard eight-channel head coil. With a slice thickness = 3 mm and a matrix = 256 × 256, the following sequences were performed: axial T1 (TR/TE = 475/15 ms), axial T2 (TR/TE = 1250/100 ms), and axial FLAIR (TR/TE/TI = 8000/140/2000 ms). For the contrast-enhanced T1-MR, post-contrast axial images were acquired after administration of gadolinium-based contrast agents with a dose of 0.1 mmol/kg. The axial DW-MR were performed with multi-section single-shot spin-echo planar imaging (EPI) sequence (TR/TE/NEX = 3000/88/1) using *b*-values of 0 and 1000 $s/{\mathrm{mm}}^{2}$. It is worth-mentioning that for all modalities, multiple axial cross-sections were obtained to cover the whole volume and were stored in DICOM format.

Reference Standard Diagnosis: Biopsy remains the reference standard to definitively diagnose gliomas and give a prognosis to determine and guide a treatment/management plan by pathologically testing the abnormal tissues within the brain. In most of the included subjects, excisional biopsy was performed. Multiple cores from the solid enhancing portion of the excised tumor were obtained. This solid portion differs from one tumor to another, and hence the number and locations of the cores differ according to the anatomical position of the lesion which correlates with tumor type and prognosis. For subjects with gliomas in hard-to-reach and/or sensitive areas within the brain that might be damaged by a more extensive procedure, a stereotactic needle biopsy was done. During this, a neurosurgeon drilled a small hole into the skull to allow the insertion of the thin needle. Small pieces from the abnormal tissues were then removed through the needle guided by radiological scanning. In both cases, the extracted specimens are sent to be pathologically tested, then the highest pathological glioma grade was assumed for the whole tumor.

It is worth mentioning that this study was conducted according to the guidelines of the Declaration of Helsinki and approved by the Institutional Review Board of Mansoura University (MD.20.01.278).

**Figure 2:**
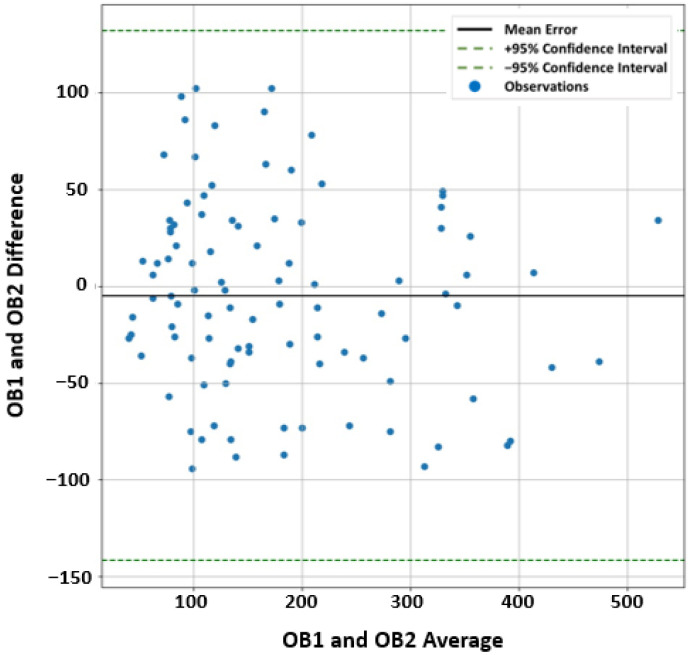
Bland-Altman mean-difference plot showing the agreement between the manual segmentation (extracted ROIs) performed by two observers (OB1 and OB2) blinded-from each other.

Glioma Tumor Preprocessing: The more accurate the segmentation is, the more precise feature extraction will be. The initial data for each subject are multiple gray-level images at different modalities stored in DICOM format. These DICOM images were transferred to a workstation (extended MR Workspace release 2.6, Philips Medical Systems, B.V., Eindhoven, The Netherlands). For each subject, regions of interest (ROIs) were manually segmented as binary masks. In the case of the T1-MR and DW-MR; the ROIs identify the tumor itself, while for the T2-MR (FLAIR), the ROIs identify the tumor with its surrounding edema. The segmentation process was performed using an in-house software by two radiologists (blinded from each other while performing the segmentation) with more than 10-years of hands-on experience in analyzing medical images. To generate the ground truth segmentation, the common area between the two observers were extracted, and then an expert radiologist with more than 25-years of hands-on experience in medical image analysis decided whether the difference should be considered as a part of the tumor or normal tissues. Meanwhile, to assess and quantify the agreement between the two segmentations, we performed Bland-Altman analysis [34]. The analysis showed a high agreement between the two segmentations with a mean absolute error of 40.556 and (−141.705, 131.867) ± 95% confidence interval. The mean-difference plot for the analysis is shown in Figure 2. Finally, 3D glioma objects were constructed and used for extracting distinguishing features.

## 3. Methods

The proposed GG-CAD system (Figure 1) can discriminate between different grades of gliomas by performing the following steps: (i) extracting higher order 3D-appearance features from the segmented glioma tumor by constructing the histogram of oriented gradients (HOG) and estimating the tumor volume from both contrast-enhanced T1-MR and from T2-MR (FLAIR), (ii) calculating first and second order textural features from the segmented tumor based on constructing histogram, gray-level co-occurrence matrix (GLCM), and gray-level run length matrix (GLRLM) by utilizing contrast enhanced T1-MR and T2-MR (FLAIR) imaging, (iii) estimating functional features by estimating 3D ADC maps for the segmented tumor from DW-MR imaging acquired at the *b*-value of 1000 $s/{\mathrm{mm}}^{2}$ and calculating the amount of enhancement between pre- and post-contrast phases of T1-MR imaging, (iv) performing features selection using Gini impurity approach over the integration of all the aforementioned extracted features to get the most significant set of features, and (v) feeding the optimal set of features to a multi-layer perceptron artificial neural networks (MLP-ANN) classification model toward getting the final diagnosis of the tumor as Grade I, II, III, or IV.

### 3.1. Engineering Features

After preprocessing glioma tumors, the structured objects which represent the different subjects should be expressed as distinguishing, standardized, and machine-understandable features. These features have the ability to discriminate between different subjects through showing the learning algorithm how to interpret the characteristics of each object. The quality of these characteristics defines and enhances our machine learning model predictive abilities. Hence, after consulting the medical team, we had agreed on multiple categories of distinctive features that suit the nature of our problem. Below, we are going in depth with the extracted imaging features.

Higher order 3D-Appearance Features: To obtain a sensitive and specific enough GG-CAD system with the ability to differentiate between different grades of glioma tumors, distinctive parametric higher order appearance features were identified. The motivation for using these 3D-appearance features relies on the hypothesis that gliomas with higher grades have more aggressive growth rates, more complex, rough, and irregular shapes than those with lower grades. Therefore, accurate identification, modeling, and extraction of such descriptors is essential towards a precise diagnosis. In the proposed framework, we identified gliomas by HOG as well as the total tumor volume.

**Figure 3:**
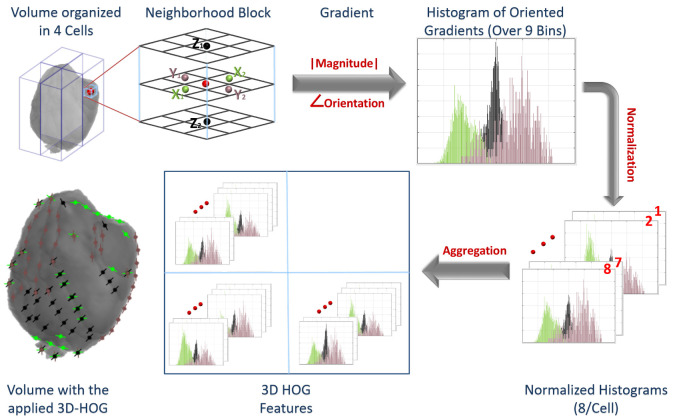
Applying the 3D HOG approach.

3D-HOG: The HOG descriptor is concerned with an object’s morphological structure/appearance finding a simplified image representation that only includes the most significant details about the image [35,36]. The HOG descriptor measures how frequent a gradient orientation occurs in a specific area of an object. In the proposed work, we applied a 3D-HOG approach over the volumes from the T2-MR (FLAIR) and pre- and post-contrast T1-MR. The extracted ROIs are resized to a new shape of (32 × 32 × Number of slices per volume). So, all the volumes have the same length (X) and width (Y), while the depth (Z) is the different dimension depending on the number of 2D slices of each volume. The resized ROI is passed to Algorithm 1 with the number of bins set to 9 and the number of cells set to 4. Finally, the HOG descriptor is formed. We have 4 cells, each cell has 8 histograms, and each histogram is generated over 9 bins. Hence, the total number of features are 4×8×9=288 features for each volume. Figure 3 shows the different steps to applying the 3D-HOG approach.
**Algorithm 1** Histogram of oriented gradients
**Input**: ROI *V* of size m×n×p and voxel size Δx×Δy×Δz, cell size *C* and
             number of bins *B*

**Output**: The HOG $H_{ijk}$**_1_****foreach ***voxel (i,j,k)*** do****_2_**($\;\;\;M_{ijk}:=\sqrt{{\left(V_{i+1,jk}-V_{i-1,jk}\right)}^{2}+{\left(V_{ij+1,k}-V_{ij-1,k}\right)}^{2}+{\left(V_{ijk+1}-V_{ijk-1}\right)}^{2}}$**_3_**$\;\;\;\Theta_{ijk}:=\cos^{-1}\left(\frac{V_{i+1,jk}V_{i-1,jk}+V_{ij+1,k}V_{ij-1,k}+V_{ijk+1}V_{ijk-1}}{\sqrt{V_{i+1,jk}^{2}+V_{ij+1,k}^{2}+V_{ijk+1}^{2}}\sqrt{V_{i-1,jk}^{2}+V_{ij-1,k}^{2}+V_{ijk-1}^{2}}}\right)$**_4_****foreach ***non-overlapping cell i of size C×C×p*** do****_5_**(**  foreach*** bin j*** do****_6_**($\;\;\;D_{j}:=\left[\left(j-1\right)\pi /B,j\pi /B\right)$**_7_**$\;\;\;H_{ij}:=\Sigma \left\{M_{rst}|\left(r,s,t\right)\in i\wedge \Theta_{rst}\in D_{j}\right\}$**_8_****    foreach*** k in the 8-neighborhood of i*** do****_9_**($\;\;\;\;H_{ijk}:=H_{ij}/\Sigma \left\{M_{rst}|\left(r,s,t\right)\in k\right\}$

**Figure 4:**
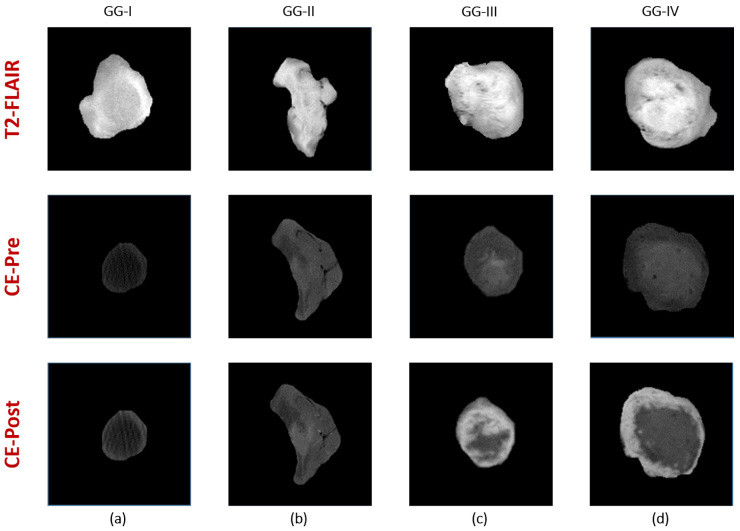
Visualization of texture differences between four gliomas with different grades (**a**) Grade I, (**b**) Grade II, (**c**) Grade III, and (**d**) Grade IV using contrast-enhanced T1-MR (pre and post phases) and T2-MR (FLAIR).

Textural Features: To enhance the performance of early differentiation between different grades of glioma tumors, we extracted comprehensive first and second order texture features that give precise description for the heterogeneity/homogeneity of the detected gliomas. We were motivated by the hypothesis that high grade glioma tumors are more heterogeneous than low grade gliomas in terms of textural appearance [37,38]. An illustrative Figure (see Figure 4) showing the texture differences between 4 subjects with different glioma grades demonstrates the feasibility of our hypothesis.

All first order textural features were estimated using a normalized empirical histogram. While first-order textural qualities are useful descriptors, they are susceptible to noise. To grant a better quantification of heterogeneity between different grades of tumors, second order textural features (GLCM and GLRLM) were used [39,40].

GLCM: The GLCM is used to evaluate the spatial interactions between voxels in a neighborhood block (reference and nearby voxels). The GLCM is a bivariate histogram encoding the frequency of neighboring voxel pairs having specific intensities. To construct the GLCM it is first necessary to identify the intensity range of the region of interest and define the level of quantization. Next, one defines the neighborhood system by specifying the spatial relationship between pairs of voxels that are considered neighbors. Each element (i,j) of the GLCM is proportional to the instance of a voxel with intensity *i* having a neighboring voxel with intensity *j*, and the matrix is normalized such that all elements sum to unity [39,41,42]. For our application, voxel intensities were 8-bit quantized, producing a 256×256 GLCM. Each voxel’s neighbors were all those within $\leq \sqrt{2}$ mm distance, so the resulting GLCM was approximately symmetric, except for the effect of boundary voxels.

GLRLM: Aside from determining the occurrence frequency of GLCM-represented voxel pairs, GLRLM examines voxel runs to determine the connectivity of voxels. GLRM encodes the number of times a run of *n* consecutive voxels occurs with the same intensity. As with the GLCM, we first define the gray level range and its quantization (0,…,255 in 8 bits), which fixes the number of rows in the GLRLM. The column dimension is the maximum extent of the region of interest in the dimension where runs are being observed. Then element (i,j) of the GLRLM is the relative instance of a run of *j* consecutive voxels all having intensity *i* [40,41,42]. Because of the difference between slice spacing and pixel spacing in the MRI data, we constructed one GLRLM for voxel runs in the *x* and *y* directions, within the same MRI slice, and another for runs in the *z* direction, i.e., across slices at the same (x,y) location in each slice. See Table A1, Table A2, Table A3, Table A4 and Table A5 for a detailed description and formulas of the extracted textural features.

Functional Features: Functionality of gliomas has direct effects on the imaging which can lead to enhancing the abilities of the model in identifying the grade of glioma tumors. Therefore, we investigated two different functional features from T1-MR and DW-MR to capture the functionality aspects of different glioma tumors.

Contrast-enhancement slopes (T1-MR): Hyperenhancement (contrast-enhancement) can characterize the functionality of a given glioma tumor. The contrast-enhancement slope can be estimated between pre- and post-contrast phases of T1-MR. The enhancement leads to remarkable changes in the gray values. These temporal changes enable the construction of contrast-enhancement slope. This slope is calculated by getting the gray-level intensity change rate over the time period between the two phases [43,44]. HGG tumors might demonstrate higher and faster slopes than those of LGG tumors.

**Figure 5:**
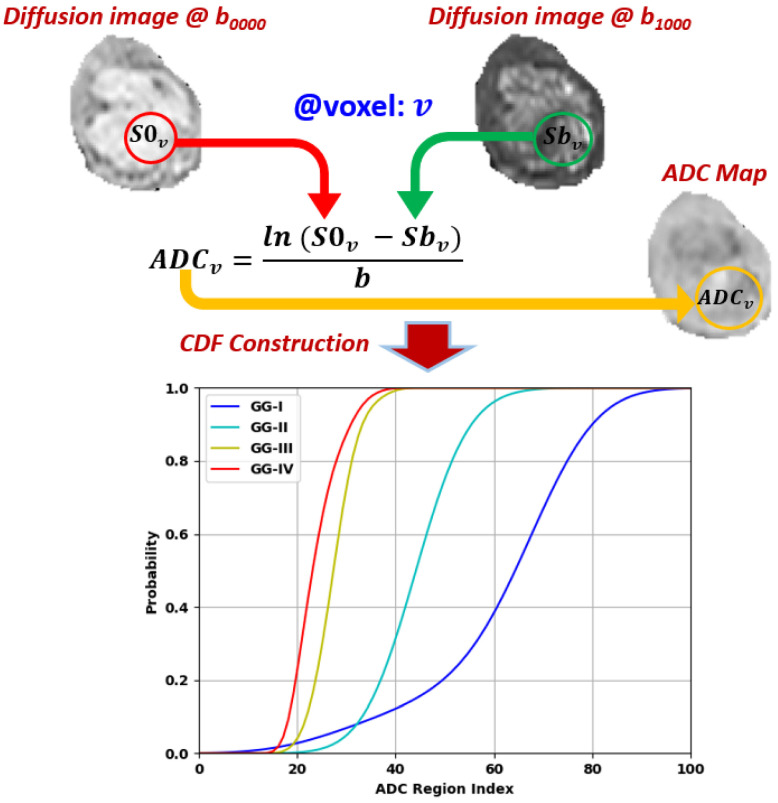
Calculations of voxel-wise apparent diffusion coefficients (ADC) for gliomas and the cumulative distribution functions (CDFs) of four gliomas with different grades.

ADCs (DW-MR): The diffusion-weighted MR signal is affected by diffusion of water through the tissue and also by capillary perfusion. Pathological tissues, such as gliomas, are likely to have unusual diffusivity properties relative to surrounding healthy tissue. Hence, well-known functional parameters called apparent diffusion coefficients (ADCs) can be estimated from different gradient field strength and duration (*b*-value). Higher *b*-values produce greater attenuation of the DW-MR signal intensity. The voxel-wise ADC values [45] at a certain *b*-value can be calculated as shown in Figure 5. Subsequently, we set the *b*-value to 1000 $s/{\mathrm{mm}}^{2}$ and calculated ADC accordingly for each voxel within the segmented tumor. Using this set of ADCs directly as a descriptive feature is problematic, since every segmented region includes a different number of voxels. Therefore, we standardized the length of the descriptor by binning the ADC measurements and calculating their cumulative distribution function, or CDF (Figure 5).

### 3.2. Classification and Hyper-Parameters Tuning

After deciding on the extracted discriminating features: appearance, textural, and functional for all glioma tumors from three different MR modalities, namely; contrast-enhanced T1-MR, T2-MR (FLAIR), and DW-MR, we proceeded with the classification process to differentiate between the different glioma grades by utilizing MLP-ANN classification models.

MLP-ANNs are well known machine learning classifiers that mainly include three types of layers: an input layer, one or more hidden layers, and an output layer, each with a large number of activation/processing units called nodes/neurons. The network is structured assuring that a full connection occurs between adjacent layers. The MLP-ANN can partition the input space of a feature into arbitrarily complex regions using non-linear activation functions used by neurons. In order to minimize the loss function, the MLP-ANN primarily employs a supervised backpropagation learning technique, where the connection weights and additive biases can be updated in the training phase using gradient descent methods [46].

Hyper-Parameters Tuning: To obtain the optimal set of MLP-ANN hyper-parameters, an in-depth grid search algorithm was employed. In the grid search, a grid containing different combinations of hyper-parameters is set up and then the MLP-ANN is trained/tested on each of the possible combinations searching for the best diagnostic performance. During the search process the accuracy of diagnosis was used as a metric for optimization purposes. In order to avoid using some hyper-parameters leading to a good performance on the training data but not so good with the test data, the hyper-parameters optimization is implemented using leave-one-subject-out (LOSO) cross-validation on all of the extracted features from all subjects within the dataset. Finally, the following parameters were approved for the proposed GG-CAD system using MLP-ANN (trainfcn: trainlm, max epochs = 500, hidden layers: hl${}_{1}$ = 200, hl${}_{2}$ = 100, hl${}_{3}$ = 50, goal = 0, max validation failure = 6, min gradient = 10${}^{-7}$, training gain (μ): initial μ = 0.001, μ decrease factor = 0.1, μ increase factor = 10, $\mu_{\max}=10^{10}$).

### 3.3. Engineering Features Selection

Typically, a features selection approach is utilized to select the optimal relevant features from a wide cohort of prospective features. This technique yields *m* features from a set of *n* options, where m<n, and *m* is the smallest set of significant and important features. Here, we applied a Gini impurity-based selection [41].

Gini Impurity-based Selection: Besides their reputation as one of the robust machine learning classifiers, random forests can be used as feature selectors. This is due to the fact that random forests’ tree-based techniques naturally rely on how effectively the purity of the node is strengthened. This indicates an impurity deterioration across all trees, which is called Gini impurity. The nodes with the highest drop in impurity are found at the beginning of the trees, while the nodes with the lowest impurity drop are found at the end. By trimming trees below a certain node or a certain impurity threshold, an optimal subset of the most significant features can be selected. The process of adopting this selection strategy are shown in Algorithm 2. To discover the best set of features to employ for the learning process, we used the Gini impurity-based technique on the entire set of the integrated features. Here, searching for the optimal impurity threshold value to be used, we applied three alternative scenarios for the selection process through using three different values of impurity threshold. Using LOSO cross-validation approach, each reduced features set is utilized (trained/tested) on the MLP classification model. Then, we compared the diagnostic performance of the developed GG-CAD system using all the extracted features with using the three reduced sets of features. From Table 1, we can find that using an impurity threshold of 0.001 gave the best performance and that a set of 332 selected features is the optimal set to be used for the proposed model. Table 2 shows the number of selected components from each group of features. Details of the selected features for the final model are shown in Table A6.
**Algorithm 2** Gini-Impurity-based Selection Process 1 Prepare the integrated features set. 2 Model and train a Random Forests classifier. 3 Set the impurity threshold. 4 Determine the features with the highest significance. 5 Build the final set that includes only these selected features.

### 3.4. Experiments

To validate the performance of the proposed GG-CAD system, the following experiments were performed:

Experiment I: To assess the final diagnostic performance of the proposed GG-CAD system, the optimal subset of features that was selected using the Gini impurity approach, were fed to a hyper-tuned MLP-ANN classification model.

It is worth mentioning that the diagnostic performance of the GG-CAD system was evaluated using a LOSO and *k*-fold (with *k* = 5 and 10) stratified cross-validation approach on the aforementioned data. For the LOSO, all data except one subject are used to train the classification model. Before the next iteration, the classification model is reinitialized, and the observation that was previously left out of the training data is included in the training data, leaving the next subject out for testing purposes. This method is performed 99 times (the included subjects in the study), with the training and testing samples being 98 and 1, respectively, at each iteration. For the *k*-fold stratified cross-validation, a subset $\frac{1}{k}\times 100\%$ of the data is chosen at random and saved for testing, while the rest ($\frac{1-k}{k}\times 100\%$) is utilized for training. In the next iteration, the classification model is reinitialized, and the subjects from the previous iteration are included in the training, leaving the next $\frac{1}{k}\times 100\%$ group of subjects for testing. This procedure is performed *k* times.

Stratification was important to eliminate any opportunity for bias and variance during the execution of *k*-fold cross-validation. The stratification technique not only achieves randomization, but it also assures that original distribution of subjects over different classes in the overall data set is maintained in the training/testing sets. To achieve stratification in our case, 13% of the training/testing sets were derived from Grade I, 22% from Grade II, 22% from Grade III, and 43% from Grade IV. These randomly stratified *k*-fold cross-validation approaches (with *k* = 5 and 10) were utilized to ensure the that the proposed model is robust and not prone to overfitting.

Experiment II: To show the added value of each group of extracted features and highlight the importance of the features integration process, we measured monothetic classifier performance using each individual group of features in turn.

Experiment III: Finally, to appreciate the diagnostic performance obtained by the developed GG-CAD system, we applied two different approaches from the literature [20,22] on our dataset (N = 99) and with the intended classification problem of glioma grading (GG-I vs. GG-II vs. GG-III vs. GG-IV) for a fair comparison. Then, we compared the final diagnostic results obtained by the developed GG-CAD system with those obtained by the two different approaches.

Evaluation Metrics: Using quadratic-weighted Cohen’s kappa [47] and accuracy as the evaluation metrics during the classification, all experimental results (Section 4) were documented in terms of mean ± standard deviation over 15 times of repetition.

## 4. Results

**Figure 6:**
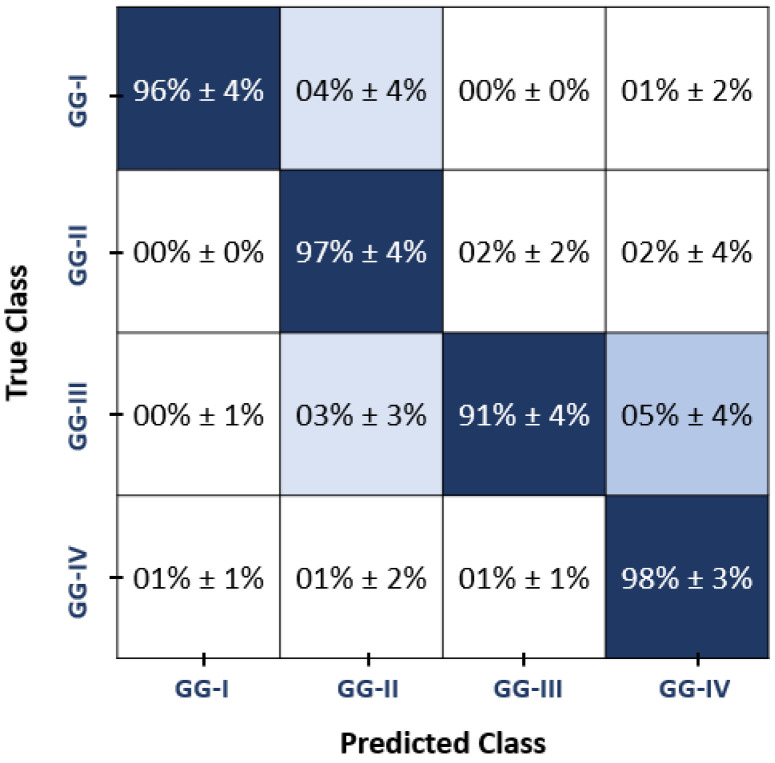
Confusion matrix of the final proposed MLP-ANN model, showing the accuracy confusion between different grades. Let GG-I, GG-II, GG-III, and GG-IV represent the four glioma grades.

Experiment I: To evaluate the classification abilities of the hyper-tuned MLP-ANN model utilized in the developed GG-CAD system, the diagnostic results using the selected optimal set of features along with MLP-ANN were obtained. To demonstrate the generalization ability and reproducibility of the GG-CAD model, a favorable comparison is performed using the three aforementioned validation approaches. As documented in Table 3, the proposed GG-CAD system shows high and robust diagnostic performance using the three different validation approaches. The confusion matrix (in terms of accuracy) of the proposed model (utilizing MLP-ANN uisng LOSO cross-validation over the selected features) is shown in Figure 6.

Experiment II: The performance of the GG-CAD system was then evaluated using the individual features along with hyper-tuned MLP-ANN classification models. To highlight the advantage of integrating these individual features, we compared the diagnostic performance of the proposed GG-CAD model (fusion of extracted features) with these individual models. With a kappa of 0.96 ± 0.02 and an overall accuracy of 95.8% ± 1.9%, the diagnostic performance of the GG-CAD system using the integrated model outperformed all other individual classification models as documented in Table 4. This can be justified by noting that integrating different quantitative features characterizes different aspects of the tumor such as, appearance, texture, and functionality.

Experiment III: Finally, to highlight the advantages of the developed GG-CAD system, in-depth comparisons with different approaches have been performed. As documented in Table 5, the diagnostic performance of the developed CAD system outperformed both the aforementioned approaches for glioma tumor grading.

## 5. Discussion

In adults, gliomas have the highest rates of prevalence and incidence among brain tumors and cause significant mortality and morbidity. They can occur anywhere in the central nervous system but primarily occur in the brain and arise in the glial tissue namely astrocytes, oligodendrocytes, and ependymal cells. Based on histological characteristics, the WHO traditionally classifies gliomas into four different grades. Grade I are solid and non-infiltrative tumors (pilocytic astrocytomas), while grades II–IV are diffuse infiltrating gliomas. Each grade of the glioma has a different treatment plan, which increases the importance of accurate grading of gliomas. Despite its demerits, surgical biopsy remains the gold standard for grading gliomas. Therefore, studying the potential for MRI-based grading of gliomas using CAD systems as a replacement for biopsy has been largely investigated with an ongoing interest [16,17,18,19,20,21,22,23,24].

In this study, the proposed GG-CAD system showed high diagnostic abilities in differentiation between different grades of glioma tumors. This precise tumor grading can guide neuro-oncologists to a proper decision for tumor treatment planning and better prognosis. Conventional MRI can assess the anatomy of tumor and advanced MRI modalities can provide quantitative assessment of tumor metabolic features, tumor cellularity, and vascularity. Hence, three different MR imaging modalities, namely; contrast-enhanced T1-MR, T2-MR (FLAIR), and DW-MR were utilized to provide different aspects of features. Using the most significant sets of the extracted discriminating features along with one of the most powerful machine learning classification techniques like MLP-ANN is proven to be highly efficient in determining the malignancy grade of a given glioma tumor.

In this work, the extracted ROIs from the images of different modalities were stacked and rendered in 3D objects representing the subjects. These 3D objects comprise multiple voxels representing the tumor itself (in contrast-enhanced T1-MR and DW-MR) or the tumor and its surrounding edema (in T2-MR (FLAIR)). Numerous histopathological parameters control the signal strength of each voxel displaying a specific gray-scale value. As a result, 3D arrays of gray-scale values in ROIs may reveal sophisticated geometric patterns that are specific to tumor grades, even if they are visually unrecognizable.

Hence, texture analysis was performed in our research. Texture analysis gives an effective description of how the gray-level of each voxel in a particular area affects the overall distribution of the values of voxels. These texture data have shown a significant impact on the performance of classification systems in a variety of studies [16,17,18,20,21,24].

The developed model utilized first and second order texture features using different methods and algorithms of texture analysis. First-order features or histogram-based features express the histogram representing the distribution of image intensity, which describe how the intensity signals of voxels are distributed over the tumor. So, these features neglect the spatial orientation and relationship between voxels. Second-order features are statistical relationships between adjacent voxels, or groups of voxels, in terms of intensity levels. Intratumoral heterogeneity is quantified by these features. These features are derived from quantitatively describing matrices encoding precise spatial relationships between voxels in a specific area in the tumor. We’ve incorporated both GLCM and GLRLM in our research [39,40]. While the GLCM shows how frequently two intensity levels appear in adjacent voxels within the tumor’s object, the GLRLM encodes the size of homogeneous runs in the same object.

In addition to the role of texture analysis in specifying the grade of gliomas, the severity of a glioma determines the 3D-appearance and shape of both the tumor and the surrounding edema. Higher grade gliomas appear to be much more complex and have sharper edges than these lower grade gliomas. This motivated us to measure 3D-appearance features to capture the potential shape differences between different grades of gliomas. Additionally, the volumes of the tumor itself and the tumor with the surrounding edema are also calculated.

One of the most beneficial aspects was to capture the functionality of different grades of gliomas that might be a key point towards achieving our goal. Functionality of gliomas has an effect on the imaging which can lead to enhancing the abilities of the model towards identifying the grade of glioma tumors. 3D DW-MR of each glioma was acquired using b=0 and b=1000 s/mm${}^{2}$). ADCs at the nonzero *b*-value were then calculated to capture the functionality differences between subjects with different grades. In contrast-enhanced T1-MR, the contrast-enhancement changes which differ according to the severity of the tumor and this difference is also captured. Contrast-enhancement slopes detecting the changes in gray-level distribution between pre- and post-contrast phases are estimated to quantify the enhancement variations between the different grades.

In the classification process, we incorporated all of the features with machine learning models. Most of these characteristics differed significantly amongst glioma grades, however there was still a significant overlap. Even when the most appropriate MR sequence has been utilized, such diversity negatively affects the ability of using a single feature class to accurately identify the glioma grade. On the other hand, using an optimal set of significant features selected from the combination of all features provided a better feature representation to detect the grades of different gliomas. With high classification performance, the developed GG-CAD system discriminated between the different grades of gliomas using the optimal set of features selected from the integration of all extracted features. The obtained results demonstrate the diagnostic abilities of the proposed model as well as the clinical utility of our methods when combined with MR imaging in the computer-aided diagnosis of brain malignancies. These results are reported in Table 1, Table 3, Table 4 and Table 5. Moreover, Figure 6 shows the confusion matrix of the final proposed model.

## 6. Conclusions and Future Work

To sum up, the developed GG-CAD system integrates and selects the optimal appearance, textural, and functional features, which demonstrated an impressive diagnostic performance (kappa = 0.96 ± 0.02 and overall accuracy = 95.8% ± 1.9%) using a MLP-ANN classification model. The viability of integrating different significant features representing diverse elements of the glioma tumor characteristics, namely; appearance, texture, and functionality is demonstrated by these findings. This study, however, is limited by the relatively small data size. We are currently collecting a larger data cohort to investigate the abilities of different deep learning (e.g., CNN and stacked-autoencoders) in segmenting glioma tumors, extracting best discriminating features, and identifying the glioma grade in a fully-automated way. Having a larger cohort of data will also enable us to study the effect of different patients’ age groups on the precise grading of gliomas. Our future work will include correlating the pathological diagnosis with the treatment response (e.g., no response, partial response, complete response, or progressive response). The success of such findings will lead to building a computer-aided prediction (CAP) system that will be able to predict the treatment response in an objective way to identify the best treatment plan for each glioma patient. That is known as a personalized medicine treatment plan.

## Figures and Tables

**Table 1:** Diagnostic performance (in terms of quadratic-weighted Cohen’s kappa and accuracy of the proposed GG-CAD system using the integrated features vs. three different sets of selected features. Let *m*, IT, GG-I, GG-II, GG-III, and GG-IV denote the number of features, impurity threshold, Glioma Grades I, II, III, and IV, respectively.

Selection	*m*	Kappa	Accuracy%				
			GG-I	GG-II	GG-III	GG-IV	Overall
All features	1099	0.94 ± 0.04	95.4 ± 5.5	95.8 ± 4.2	89.4 ± 5.7	96.4 ± 1.5	94.6 ± 2.3
IT = 0.0010	332	0.96 ± 0.02	95.9 ± 3.8	96.7 ± 4.2	91.2 ± 4.2	97.6 ± 2.5	95.8 ± 1.9
IT = 0.0015	128	0.90 ± 0.08	87.7 ± 7.3	91.8 ± 6.3	90.0 ± 6.0	97.5 ± 2.7	93.3 ± 3.9
IT = 0.0020	61	0.86 ± 0.04	81.5 ± 8.8	87.3 ± 3.4	87.6 ± 7.1	94.9 ± 3.2	89.8 ± 3.0

**Table 2:** Extracted features taxonomy with their various types and counts before and after features selection. Let *n* and *m* denote number of features before and after selection, respectively.

Features	MR Modality	*n*	*m*
Higher order 3D-Appearance Features			
3D-HOG + Volume	T2-MR (FLAIR)	289 (288 + 1)	82 (81 + 1)
3D-HOG	T1-MR (Pre-contrast)	288	58
3D-HOG + Volume	T1-MR (Post-contrast)	289 (288 + 1)	46 (45 + 1)
Textural Features			
Histogram + GLCM + GLRLM	T2-MR (FLAIR)	44 (26 + 6 + 12)	30 (18 + 4 + 8)
Histogram + GLCM + GLRLM	T1-MR (Pre-contrast)	44 (26 + 6 + 12)	35 (21 + 5 + 9)
Histogram + GLCM + GLRLM	T1-MR (Post-contrast)	44 (26 + 6 + 12)	37 (22 + 6 + 9)
Functional features			
CDFs of ADCs ($b_{1000}$ $s/{\mathrm{mm}}^{2}$)	DW-MR	100	43
Contrast-enhancement Slope	T1-MR	1	1
Integrated Features			
Integrated	All	1099	332

**Table 3:** Diagnostic performance (in terms of quadratic-weighted Cohen’s kappa and accuracy) of the proposed GG-CAD system using a LOSO and *k*-fold (with *k* = 5 and 10) stratified cross-validation approach on the optimal set of features. Let GG-I, GG-II, GG-III, and GG-IV denote Glioma Grades I, II, III, and IV, respectively.

Approach	Kappa	Accuracy%				
		GG-I	GG-II	GG-III	GG-IV	Overall
LOSO	0.96 ± 0.02	95.9 ± 03.8	96.7 ± 04.2	91.2 ± 04.2	97.6 ± 02.5	95.8 ± 1.9
10-Fold	0.91 ± 0.02	94.9 ± 06.1	93.0 ± 03.3	87.0 ± 05.7	95.2 ± 01.5	92.9 ± 01.8
5-Fold	0.90 ± 0.03	89.7 ± 06.1	93.6 ± 05.5	80.9 ± 05.3	93.7 ± 01.7	90.3 ± 02.4

**Table 4:** Diagnostic performance (in terms of quadratic-weighted Cohen’s kappa and accuracy) of the proposed GG-CAD system using the integrated features vs. individual ones using MLP-ANN. Let GG-I, GG-II, GG-III, and GG-IV denote Glioma Grades I, II, III, and IV, respectively. The MLP-ANN column shows the structure/architecture of the MLP-ANN models.

MR Modality	Kappa	Accuracy%					MLP-ANN
		GG-I	GG-II	GG-III	GG-IV	Overall	
Higher order 3D-Appearance Features							
T2-MR (FLAIR)	0.85 ± 0.03	93.3 ± 7.4	84.6 ± 8.4	73.0 ± 6.7	94.4 ± 2.4	87.3 ± 2.2	(50, 25)
T1-MR (Pre)	0.71 ± 0.06	83.6 ± 7.4	79.7 ± 5.5	67.6 ± 3.7	92.9 ± 1.7	83.1 ± 1.9	(50, 25)
T1-MR (Post)	0.78 ± 0.05	87.7 ± 3.8	78.2 ± 6.0	66.1 ± 4.9	93.3 ± 1.3	83.2 ± 1.6	(50, 25)
Textural Features							
T2-MR (FLAIR)	0.82 ± 0.02	87.7 ± 7.3	72.1 ± 6.0	60.9 ± 4.9	93.7 ± 2.2	80.8 ± 1.2	(25, 10)
T1-MR (Pre)	0.79 ± 0.04	79.5 ± 8.7	76.7 ± 6.4	63.9 ± 5.6	91.6 ± 2.4	80.5 ± 1.8	(25, 10)
T1-MR (Post)	0.78 ± 0.05	86.2 ± 4.2	81.2 ± 2.3	70.3 ± 6.0	92.2 ± 1.1	84.1 ± 2.5	(25, 10)
Functional Features							
DW-MR & T1-MR	0.79 ± 0.05	82.6 ± 7.7	78.8 ± 5.2	64.6 ± 6.5	91.8 ± 1.7	81.6 ± 2.5	(50, 25)
Integrated Features							
GG-CAD (All)	0.96 ± 0.02	95.9 ± 3.8	96.7 ± 4.2	91.2 ± 4.2	97.6 ± 2.5	95.8 ± 1.9	(200, 100, 50)

**Table 5:** The final diagnostic performance (in terms of quadratic-weighted Cohen’s kappa and accuracy) for grading the tumors into (GG-I, GG-II, GG-III, and GG-IV) by using (a) the proposed CAD system, (b) approach by Alis et al. [20], and (c) approach by Kalaiselvi et al. [22].

Model	Kappa	Accuracy%				
		GG-I	GG-II	GG-III	GG-IV	Overall
GG-CAD	0.96 ± 0.02	95.9 ± 03.8	96.7 ± 04.2	91.2 ± 04.2	97.6 ± 02.5	95.8 ± 01.9
Alis [20]	0.79 ± 0.05	62.1 ± 16.5	77.3 ± 06.4	66.7 ± 02.1	90.5 ± 01.9	78.5 ± 04.6
Kalaiselvi [22]	0.82 ± 0.05	77.4 ± 15.0	78.8 ± 05.4	67.0 ± 08.2	90.5 ± 02.1	80.9 ± 03.5

## Data Availability

Data could be made available after acceptance upon a reasonable request to the corresponding author.

## Appendix A

In this appendix, we are going to detail the mathematical formulas used to extract the textural features and the definitions of these features are also given. Therefore, the following basic notations will be used:

Notation

- N: The largest possible run length.
- $N_{p}$: Gray-level intensity levels.
- $N_{g}$: Normalized intensity levels.
- $n_{r}$: The overall count of runs.
- $n_{p}$: The overall count of pixels.
- (i,j): Row and column index, respectively.
- P(i): Gray-level intensity values.
- g(i): Normalized gray-level intensity values.
- ϵ: Arbitrarily small, positive number
- GLCM: The gray-level Co-occurrence Matrix.
- CM: The normalized GLCM.
- CM(i,j): Element at position (i,j) in the normalized GLCM.
- RL: The gray-level Run Length Matrix.
- RL(i,j): Element at position (i,j) in the GLRLM.
- $\mu_{x}$: The marginal rows mean.
- $\mu_{y}$: The marginal columns mean.
- $\sigma_{x}$: The marginal rows standard deviation.
- $\sigma_{y}$: The marginal columns standard deviation.

**Table A1:** First order textural features.

Textural Feature	Definition
Mean (μ)	Average MR signal intensity (T1-weighted or T2-weighted) of the object of interest.
Variance	Second central moment of MR signal intensity distribution.
Skewness	Asymmetry of signal intensity distribution.
Kurtosis	Tail weight of signal intensity distribution.
Entropy	Metric of randomness of the intensity distribution.
CDFs	Cumulative sum of the relative frequency histogram.
Percentiles	Inverse function of the CDF.

**Table A2:** Second order textural features.

Textural Feature	Definition
GLCM	
Contrast	Average intensity difference between a voxel and its neighbors.
Dissimilarity	Extent to which neighboring voxels differ in intensity.
Homogeneity	Large-scale uniformity of voxel intensities.
Angular second moment (ASM)	Local uniformity of voxel intensities.
Energy	The square root of the ASM.
Correlation	Linear dependency between neighboring voxels.
Gray-level non-uniformity (GLN)	Dissimilarity of gray-level values within the object.
High gray-level run emphasis (HGLRE)	Incidence of runs of high intensity.
Long run emphasis (LRE)	Distribution of long runs.
Long run high gray-level emphasis (LRHGLE)	Distribution of long runs of high intensity.
Long run low gray-level emphasis (LRLGLE)	Distribution of long runs of low intensity.
Low gray-level run emphasis (LGLRE)	Incidence of runs of low intensity.
Run entropy (RE)	Degree of randomness in the run lengths.
Run length non-uniformity (RLN)	Degree of inhomogeneity in the run lengths.
Run percentage (RP)	Homogeneity of homogeneous runs.
Short run emphasis (SRE)	Distribution of short runs.
Short run high gray-level emphasis (SRHGLE)	Distribution of short runs of high intensity.
Short run low gray-level emphasis (SRLGLE)	Distribution of short runs of low intensity.

**Table A3:** First order Histogram textural features formulas.

Feature	Formula
Skewness	(A1) $\frac{\frac{1}{N_{p}}\sum_{i=1}^{N_{p}}{\left(P\left(i\right)-\mu \right)}^{3}}{{\left(\sqrt{\frac{1}{N_{p}}\sum_{i=1}^{N_{p}}{\left(P\left(i\right)-\mu \right)}^{2}}\right)}^{3}}$
Kurtosis	(A2) $\frac{\frac{1}{N_{p}}\sum_{i=1}^{N_{p}}{\left(P\left(i\right)-\mu \right)}^{4}}{{\left(\frac{1}{N_{p}}\sum_{i=1}^{N_{p}}{(P(i)-\mu )}^{2}\right)}^{2}}$
Entropy	(A3) $-\sum_{i=1}^{N_{g}}g\left(i\right)\log_{2}\left(g(i)+\epsilon \right)$

**Table A4:** Second order GLCM textural features and their associated formulas.

Feature	Formula
Contrast	(A4) $\sum_{i=0}^{N_{g}}\sum_{j=0}^{N_{g}}{\left(i-j\right)}^{2}CM\left(i,j\right)$
Dissimilarity	(A5) $\sum_{i=0}^{N_{g}}\sum_{j=0}^{N_{g}}\left|i-j|CM(i,j\right)$
Homogeneity	(A6) $\sum_{i=0}^{N_{g}}\sum_{j=0}^{N_{g}}\frac{CM(i,j)}{1+{\left(i-j\right)}^{2}}$
ASM	(A7) $\sum_{i=0}^{N_{g}}\sum_{j=0}^{N_{g}}{\left(CM(i,j)\right)}^{2}$
Energy	(A8) $\sqrt{ASM}$
Correlation	(A9) $\frac{\sum_{i=0}^{N_{g}}\sum_{j=0}^{N_{g}}CM\left(i,j\right)ij-\mu_{x}\mu_{y}}{\sigma_{x}\left(i\right)\sigma_{y}\left(j\right)}$

**Table A5:** Second order GLRLM textural features and their associated formulas.

Feature	Formula
Gray-Level Non-Uniformity (GLN)	(A10) $\frac{\sum_{i=0}^{N_{g}}{\left(\sum_{j=0}^{N-1}RL(i,j)\right)}^{2}}{n_{r}}$
High Gray-Level Run Emphasis (HGLRE)	(A11) $\frac{\sum_{i=0}^{N_{g}}\sum_{j=1}^{N-1}RL\left(i,j\right)i^{2}}{n_{r}}$
Long Run Emphasis (LRE)	(A12) $\frac{\sum_{i=0}^{N_{g}}\sum_{j=0}^{N-1}RL\left(i,j\right)j^{2}}{n_{r}}$
Long Run High Gray-Level Emphasis (LRHGLE)	(A13) $\frac{\sum_{i=0}^{N_{g}}\sum_{j=0}^{N-1}RL\left(i,j\right)i^{2}j^{2}}{n_{r}}$
Long Run Low Gray-Level Emphasis (LRLGLE)	(A14) $\frac{\sum_{i=0}^{N_{g}}\sum_{j=0}^{N-1}\frac{RL\left(i,j\right)j^{2}}{i^{2}}}{n_{r}}$
low gray-level run emphasis (LGLRE)	(A15) $\frac{\sum_{i=0}^{N_{g}}\sum_{j=0}^{N-1}\frac{RL(i,j)}{i^{2}}}{n_{r}}$
Run Entropy (RE)	(A16) $-\sum_{i=0}^{N_{g}}\sum_{j=0}^{N-1}RL\left(i,j\right)\log_{2}RL\left(i,j\right)+\epsilon )$
Run Length Non-Uniformity (RLN)	(A17) $\frac{\sum_{j=0}^{N-1}{\left(\sum_{i=0}^{N_{g}}RL(i,j)\right)}^{2}}{n_{r}}$
Run Percentage (RP)	(A18) $\frac{n_{r}}{n_{p}}$
Short Run Emphasis (SRE)	(A19) $\frac{\sum_{i=0}^{N_{g}}\sum_{j=0}^{N-1}\frac{RL(i,j)}{j^{2}}}{n_{r}}$
Short Run High gray-level Emphasis (SRHGLE)	(A20) $\frac{\sum_{i=0}^{N_{g}}\sum_{j=0}^{N-1}\frac{RL\left(i,j\right)i^{2}}{j^{2}}}{n_{r}}$
Short Run Low gray-level Emphasis (SRLGLE)	(A21) $\frac{\sum_{i=0}^{N_{g}}\sum_{j=0}^{N-1}\frac{RL(i,j)}{i^{2}j^{2}}}{n_{r}}$

**Table A6:** Details of the selected features for the final model.

Higher Order 3D-Appearance Features		
**T2-MR (FLAIR)**	81 HOG-Components + Volume	
**T1-MR (Pre-contrast)**	58 HOG-Components	
**T1-MR (Post-contrast)**	45 HOG-Components + Volume	
**Textural Features**		
**T2-MR (FLAIR)**	**Histogram**	Mean (μ), Variance, Skewness, Kurtosis, Entropy, 6 CDFs-Components, 7 Percentiles-Components
	**GLCM**	Contrast, Dissimilarity, Homogeneity, ASM
	**GLRLM**	HGLRE, LRE, LRHGLE,LGLRE, RE, RLN, SRE, SRHGLE
**T1-MR (Pre-contrast)**	**Histogram**	Mean (μ), Variance, Skewness, Kurtosis, Entropy, 8 CDFs-Components, 8 Percentiles-Components
	**GLCM**	Contrast, Dissimilarity, Homogeneity, Energy, Correlation
	**GLRLM**	GLN, LRE, LRLGLE, LGLRE, RE, RLN, RP, SRE, SRLGLE
**T1-MR (Post-contrast)**	**Histogram**	Mean (μ), Variance, Skewness, Kurtosis, Entropy, 8 CDFs-Components, 9 Percentiles-Components
	**GLCM**	Contrast, Dissimilarity, Homogeneity, ASM, Energy, Correlation
	**GLRLM**	HGLRE, LRE, LRHGLE, LRLGLE, LGLRE, RE, RLN, SRHGLE, SRLGLE
**Functional features**		
**DW-MR**	43 CDFs-Components	
**T1-MR**	Contrast-enhancement Slope	
